# Extensive Gustatory Cortex Lesions Significantly Impair Taste Sensitivity to KCl and Quinine but Not to Sucrose in Rats

**DOI:** 10.1371/journal.pone.0143419

**Published:** 2015-11-23

**Authors:** Michelle B. Bales, Lindsey A. Schier, Ginger D. Blonde, Alan C. Spector

**Affiliations:** Department of Psychology and Program in Neuroscience, Florida State University, Tallahassee, Florida, United States of America; Duke University Medical Center, UNITED STATES

## Abstract

Recently, we reported that large bilateral gustatory cortex (GC) lesions significantly impair taste sensitivity to salts in rats. Here we extended the tastants examined to include sucrose and quinine in rats with ibotenic acid-induced lesions in GC (GCX) and in sham-operated controls (SHAM). Presurgically, immediately after drinking NaCl, rats received a LiCl or saline injection (i.p.), but postsurgical tests indicated a weak conditioned taste aversion (CTA) even in controls. The rats were then trained and tested in gustometers to discriminate a tastant from water in a two-response operant taste detection task. Psychometric functions were derived for sucrose, KCl, and quinine. Our mapping system was used to determine placement, size, and symmetry of the lesions (~91% GC damage on average). For KCl, there was a significant rightward shift (ΔEC_50_ = 0.57 log_10_ units; p<0.001) in the GCX psychometric function relative to SHAM, replicating our prior work. There was also a significant lesion-induced impairment (ΔEC_50_ = 0.41 log_10_ units; p = 0.006) in quinine sensitivity. Surprisingly, taste sensitivity to sucrose was unaffected by the extensive lesions and was comparable between GCX and SHAM rats. The fact that such large bilateral GC lesions did not shift sucrose psychometric functions relative to SHAM, but did significantly compromise quinine and KCl sensitivity suggests that the neural circuits responsible for the detection of specific taste stimuli are partially dissociable. Lesion-induced impairments were observed in expression of a postsurgical CTA to a maltodextrin solution as assessed in a taste-oriented brief-access test, but were not reflected in a longer term 46-h two-bottle test. Thus, deficits observed in rats after extensive damage to the GC are also dependent on the test used to assess taste function. In conclusion, the degree to which the GC is necessary for the maintenance of normal taste detectability apparently depends on the chemical and/or perceptual features of the stimulus.

## Introduction

In rodents, the gustatory cortex (GC) is conventionally defined as a subregion of insular cortex surrounding the intersection of the middle cerebral artery and the rhinal fissure. Electrophysiological recordings have revealed taste-responsive neurons distributed in this brain region and tracing studies have confirmed projections from the gustatory zone of the thalamus [1–12]. Although the GC is highly integrated with other taste pathways, its role in taste function, including qualitative discrimination, affect, and physiological reflexes remains in its infancy.

Primary gustatory afferents terminate in the rostral nucleus of the solitary tract which sends taste input to the parabrachial nucleus (PBN) of the pons. From there, two different taste pathways emerge destined for the forebrain. A thalamocortical pathway courses from the PBN to the parvicellular region of the ventral posteromedial nucleus of the thalamus (VPMpc), and, then onto the GC. A ventral forebrain pathway projects from the PBN to various structures, including the lateral hypothalamus, bed nucleus of the stria terminalis, and the central nucleus of the amygdala [13–17]. Pfaffmann et al. [18] initially hypothesized that the thalamocortical pathway is involved in stimulus identification, while the ventral forebrain gustatory pathway is involved in taste-guided motivation and affect. Although there is substantial evidence for the latter, the former has lacked comprehensive experimental testing.

To date, the functional role of GC has mostly been studied in the context of taste memory as assessed by conditioned taste aversion (CTA), in which ingestion of a novel tastant (considered the conditioned stimulus [CS]) is followed by administration of an agent that causes visceral malaise such as LiCl (considered the unconditioned stimulus [US]). On subsequent occasions, animals that have received this training will avoid ingesting the CS. Often, bilateral damage to the GC interferes with the expression of a presurgical CTA (e.g., [3, 19–23]). Although results vary, typically postsurgical CTA acquisition is also impaired by manipulations that compromise GC function (e.g., [19–20, 22, 24–38]; (but see [39])). Recent data from this laboratory confirm that some lesions within insular cortex, including in GC, hinder CTA expression but also show that large areas of the GC can be damaged without effect [40–42]. One of these studies revealed, in fact, that bilateral lesions in a key site in insular cortex that included the posterior half of the conventionally defined GC and the adjacent posterior portions of insular cortex was associated with significant CTA expression deficits, whereas lesions in anterior portions of GC were without effect, highlighting a potential for regional specificity [41, 43].

In terms of unconditioned responsiveness to prototypical tastants, although GC lesions have been reported to raise quinine avoidance thresholds in rats under some but not all intake testing conditions [44], unconditioned preference and avoidance are largely undisturbed [20, 26, 45]. However, intake test outcomes can also be influenced by non-gustatory factors such as stimulation of postingestive receptor systems [46]. In a brief-access taste test which minimizes non-gustatory influences, rats with extensive centrally placed GC lesions had normal unconditioned responsiveness to various concentrations of sucrose and quinine [40]. Likewise, rats with large GC lesions exhibited the normal pattern of stereotypical oromotor movements in response to single concentrations of sucrose and quinine as they were infused directly into the oral cavity [47]. Overall, these intake, brief-access, and taste reactivity tests suggest that the GC is not involved in the unconditioned affective response to taste stimuli. The contribution of the GC to the sensory-discriminative aspects of taste function, independent of other stimulus characteristics, however, still remains to be thoroughly tested.

Recently, we applied a two-response operant taste discrimination task to test whether extensive bilateral damage of the GC has an impact on taste sensitivity to NaCl and KCl. By requiring immediate responses to very small volumes of a sample stimulus, this task, like the brief-access test, reduces postingestive factors. However, unlike brief-access tests, this operant task forces water-deprived rats to use taste as a discriminative cue to obtain water; accordingly, the responses are motivated by thirst and not the hedonic aspects of the tastants. This task revealed deficits in taste detection for KCl and NaCl in rats with ~80% damage to the GC on average. These same rats also had difficulty learning to discriminate between KCl and NaCl. However, higher salt concentrations could be detected, and the discrimination was eventually learned, suggesting that other central taste pathways can maintain salt taste discrimination function [42]. Here, we aimed to broaden the stimuli tested to include the prototypical sweet and bitter tastants, sucrose and quinine, to assess the effects of GC lesions on taste detection. To link our prior work to this study, we included KCl as a test stimulus for comparison. Impairments in taste detection were also compared to the effects of the GC lesion on CTA expression. In an effort to better characterize the size, location, and bilateral symmetry of the ibotenic acid-induced cortical damage, we applied a high-resolution lesion mapping system similar to that successfully employed in our prior studies [41–43, 47].

## Materials and Methods

### Subjects

Forty-four male Sprague-Dawley rats (Charles River Laboratories) weighing 317–352 g (335±18 g) at the start of the experiment served as subjects. The rats were individually housed in polycarbonate cages in a room with 12:12-h light:dark cycle that was climate-controlled. Every rat had access to a stainless steel toy, which was not ingestible, as a form of enrichment. Ad libitum chow (Rodent Diet 5001; PMI) and deionized water (DW) were provided unless noted otherwise below. During sucrose detection training and again during KCl testing through quinine training I (see below), the animals were injected with up to 0.4 mg/kg of Ivermectin (s.c.) every 3 to 6 days as part of a vivarium-wide pinworm eradication program. It is important to note that both sham-operated controls and animals with lesions were treated the same. All procedures were approved by the Animal Care and Use Committee at Florida State University (Protocol Number: 1012) and complied with the National Institutes of Health guidelines for humane handling of animals. All surgery was performed under isoflurane anesthesia, and efforts were made to minimize suffering.

### Stimuli

Taste stimuli were made with reagent-grade chemicals (except for Maltrin) and dissolved in DW fresh each day. The CS in the presurgical CTA was 0.1 M NaCl (Macron Fine Chemicals) and the CS in the postsurgical CTA was 5% MaltrinQD M580 Maltodextrin (Maltrin; Grain Processing Corporation). Several concentrations of the Maltrin were used in the brief-access taste test (1.25, 2.5, 5, 10, and 20%). Psychometric sensitivity functions were obtained for sucrose (0.0005, 0.001, 0.002, 0.003, 0.005, 0.01, 0.01875, 0.0375, 0.075, 0.15, 0.3, and 0.6 M; Macron Fine Chemicals), KCl (0.0005, 0.001, 0.0025, 0.005, 0.01, 0.02, 0.04, 0.08, 0.15, 0.3, and 0.6 M; Sigma Aldrich), and quinine hydrochloride (0.00006, 0.0002, 0.0005, 0.002, 0.006, 0.025, 0.1, and 0.4 mM; Sigma Aldrich).

### Apparatus

Training and testing for the derivation of psychometric sensitivity functions and for the brief-access postsurgical CTA assessment (see below) were conducted in a computer-controlled device referred to as a gustometer [48]. This device allows for delivery of small and relatively controlled volumes per lick (~5 μl) and the measurement of immediate licking responses. The testing chamber consists of three Plexiglas sides and a front stainless steel panel, with a wire mesh floor. The rat can have access to up to three drinking balls through slots at the front of the chamber. A borosilicate glass sample ball is accessible through the middle slot. As it rotates on a fixed axis, licks are registered with a force transducer that is secured to a motor-driven mechanical arm. The arm positions the ball between the center access slot and polytetrafluoroethylene (PTFE) tubing that is threaded through a turret. The sample stimuli are delivered through these tubes and deposited on the sample ball. To initiate stimulus delivery and to ensure the rat is actively licking, the rat must lick the sample ball twice within 250-ms to activate an infusion pump, which, in turn, delivers ~10 μl of sample stimulus that serves to coat the ball. Following the preload, ~5 μl of sample stimulus is delivered per lick. After a trial is completed, the mechanical arm moves the ball back into a cleaning well where it is rinsed with DW and dried with pressurized air during the intertrial interval. Through slots on either side of the sample ball, the rat can access two response balls that record licks with force transducers. Fluid reinforcement is delivered through tubing which is threaded through the ball. A cue light is positioned above each reinforcement ball. All three drinking slots can be manually blocked via stainless-steel shutters. The chamber sits in a sound attenuating enclosure. The enclosure has a speaker which presents a masking noise to minimize auditory cues. A ventilation system designed to minimize olfactory cues includes a duct that is attached to a wall of the enclosure and is positioned above the sample ball. To prevent visual cues, a shield is anchored in front of the stimulus delivery turret. A house light is mounted in the ceiling of the sound attenuating enclosure above the chamber.

### Surgery

Surgery was performed 20–83 days after the presurgical CTA (see below; Table 1). The rats were assigned to receive bilateral injections into the gustatory cortex of either ibotenic acid (N = 30; 20 mg/ml, dissolved in PBS) or PBS (N = 14). The groups were balanced on the basis of body weight, CS consumption, US injection and postsurgical apparatus assignment.

**Table 1:** Schedule of experimental phases.

Phase	Session Length	# of Days	Taste Stimuli
**Conditioned Taste Aversion (CTA):**			
Training	15 min	4	DW
Conditioning Trial*	15 min	1	0.1 M NaCl
**Surgery:** GCX rats given ibotenic acid lesions targeting GC and SHAM rats given PBS (SHAM) vehicle infusions			
Recovery		14–81	
CTA Retention			
Training	30 min	4	DW
Intake Test	30 min	1	0.1 M NaCl
Preference Test	23 hr	2	0.1 M NaCl and DW
**Detection Thresholds:**			
Sucrose			
Lick Training	30 min	3	DW
Side Training	30 min	6	0.6 M sucrose or DW
Alternation	30 min	4	0.6 M sucrose or DW
Random Training I	30 min	2	0.6 M sucrose or DW
Random Training II	30 min	4	0.6 M sucrose or DW
Testing	30 min	33–36	Sucrose** and DW
KCl			
Training	30 min	3	0.6 M KCl and DW
Testing	30 min	29–49	KCl** and DW
Water Test	30 min	1	DW
Quinine			
Training I	30 min	34	0.1, 0.2, or 0.4 mM quinine and DW
Testing I	30 min	20	Quinine** and DW
Water Test I	30 min	2	DW
Training II	30 min	1	0.4 mM quinine and DW
Testing II	30 min	20–23	Quinine** and DW
Water Test II	30 min	1	DW
**CTA:**			
Training	15 min	4	DW
First Conditioning Trial*	15 min	1	5% Maltrin
Training	15 min	2	DW
Second Conditioning Trial*	15 min	1	5% Maltrin
Brief-access Training	30 min	2	DW
Brief-access Testing	30 min	1	1.25, 2.5, 5, 10 and 20% Maltrin
Preference Test	23 hr	2	5% Maltrin and DW

*The unconditioned stimulus immediately followed CS presentation: 0.15 M LiCl or NaCl (2 mEq/kg, i.p.).

**One concentration was tested per session from a range of sucrose, KCl and quinine concentrations.

The rats were initially anesthetized with 5% isoflurane and then secured in a stereotaxic instrument via non-traumatic earbars and maintained in an anesthetic state with 2–3% isoflurane delivered through a nosecone. A ~3 cm midline incision exposed the skull which was leveled by adjusting the bite bar to equalize the dorsal/ventral (DV) coordinates of bregma and lambda.

Because, as conventionally defined, GC extends over 2 mm along the AP axis, two injection sites, one rostral and one caudal, were used to disperse the ibotenic acid across the entire span of GC in both hemispheres. A glass micropipette (tip diameter: ~50 μm) was fitted on the barrel of a Hamilton syringe and sealed with paraffin. Holes were drilled into the skull over the injection sites. Once the dura was exposed, it was carefully cut using a 23G needle and the micropipette was inserted through the opening. The rostral injection (0.21 μl) was positioned at +1.5 mm AP, ± 5.2 mm ML, and -6.4 mm DV and the caudal injection (0.18 μl) was positioned at +0.5 mm AP, ± 5.8 mm ML, and -6.6 mm DV, relative to bregma. Each injection began 2 min after inserting the micropipette and was removed 2 min after the injection was complete. The injection took place over 10 min to ensure the ibotenic acid was evenly diffused. After each injection, a small amount of ibotenic acid was dispensed to confirm that the micropipette had not clogged, an event which never occurred. Wound clips were used to close the incision and were removed 7–10 days after surgery. The antibiotic Procaine G penicillin (30,000 units, s.c.) and the analgesic carprofen (5 mg/kg body mass, s.c.) injections were given on the day of and three days following surgery to prevent infection and to relieve pain and inflammation.

### CTA Training and Testing

#### Presurgical CTA Training

Rats were water deprived and trained to drink DW during 15-min sessions in the mornings so they would drink the CS on the conditioning day. All of the rats were given 30-min access to DW approximately 5 h after their 15-min morning intake. The rats were placed on this restricted water-access schedule for 4 consecutive days. On the fifth day, the rats received 0.1 M NaCl (CS) during their 15-min morning intake instead of DW, followed immediately by an injection of 0.15 M LiCl (2 mEq/kg) or an equivalent volume of 0.15 M NaCl (US; i.p.). Injection groups were balanced on the basis of body weight and the last 15-min DW intake. Intake volumes were measured to the nearest 0.5 mL. DW was returned in the afternoon after the conditioning trial.

#### Postsurgical Assessment of Presurgical CTA

Once all the rats had recovered from surgery, they were water deprived and given 30-min DW in the morning and 30-min supplemental DW in the afternoon (beginning 18–81 days after surgery). After 4 days on this restricted water-access schedule, all of the rats had consumed DW during the morning session for 3 consecutive days. The postsurgical CTA test occurred on the fifth day, in which the CS (0.1 M NaCl) was given for 30 min and intake was measured. Immediately following the 30-min single-bottle intake test, rats were given two bottles, one containing DW and one containing 0.1 M NaCl (CS) to begin a 46-h preference test. After each 23-h period, intake was measured, and the bottles were refilled with fresh solution, and their position on the cage was switched to prevent side bias.

#### Postsurgical CTA Training

The conditioning of the postsurgical taste aversion occurred after the psychometric taste sensitivity functions were assessed (Table 1). Postsurgical conditioning was similar to that done presurgically except 5% Maltrin was the CS and there were two conditioning trials. After four days of the restricted water-access schedule as described above, the rats received 5% Maltrin for their 15-min morning access and were then immediately injected with either 0.15 M LiCl (2 mEq/kg) or an equivalent volume of 0.15 M NaCl (US; i.p.). Injection groups were the same as for the presurgical conditioning. The rats received water during their next two morning sessions of fluid access and then received a second conditioning trial. Any rat that did not sample at least 1 mL of the CS during the 15-min access was given a single oral infusion of CS (0.5 mL) immediately before the injection, which was only necessary for 8 LiCl-injected rats on the second trial. The home cage water bottles were replaced approximately 4.5 hours after the second conditioning trial.

#### Assessment of Postsurgical CTA

Three days after the second conditioning trial, the water bottles were removed and the next day brief-access test training began in the gustometer. The rats were trained to lick DW from the sample ball for two consecutive days while the response balls were blocked by shutters. On the first training day, the rats licked freely from the sample ball during a 30-min session. On the second training day, DW was delivered in randomized blocks of six 10-s trials. A trial began after the rat licked the sample ball twice within 250-ms to ensure active licking before a stimulus was delivered and each trial was preceded by a 5-lick DW rinse (in a maximum of 2-s). During the ~6-s interval between trials, the sample ball was rinsed with DW, dried with pressurized air, and then replaced in position for the next trial. A CTA brief-access test occurred the following day and consisted of the same trial structure as the previous training day, including the 5-lick DW rinse, but the rats received 1.25, 2.5, 5, 10, 20% Maltrin or DW delivered in randomized blocks of six without replacement. Water bottles were replaced on the home cage at least 30 min after the brief-access CTA test.

The following day, a two-bottle preference test with DW and 5% Maltrin (the CS concentration) began. Intake was measured at 23-h, and the bottles were rinsed, refilled, and their positions were switched on the home cage to prevent side bias. Intake was measured again at 46-h. Water bottles were returned after testing.

### Operant Training and Assessment of Psychometric Taste Sensitivity Functions

Psychometric taste sensitivity functions were determined for sucrose, KCl, and quinine (see Table 1) through the use of a two-response operant taste discrimination task with a modified method of limits procedure for stimulus presentation similar to the one described by Blonde et al [49] with the tastant and DW trials presented randomly. The task was carried out in the gustometer described above (see Apparatus). Water bottles were removed from the home cages on Sunday afternoon before training or testing and returned on Friday after the session for ad libitum water access over the weekend. The taste stimuli and DW were delivered as described below during a 30-min session in the gustometer each weekday. If the rats did not have a 30-min session in a gustometer or if they were below 85% of their previous ad libitum body mass, they were given 1 h supplemental access to DW on the home cage.

#### Trial Structure

The rat began a trial by licking the dry sample ball twice within 250-ms to ensure active licking before the sample stimulus was delivered. Upon active licking, the stimulus was dispensed for up to 10 licks (or 3 s). After which, the rat had a restricted period of time to respond (limited hold) to the left or right response balls to receive 20 licks of DW as reinforcement. DW reinforcement was given if the rat responded to the correct side. If the rat responded to the incorrect side or did not respond, it was punished with a time-out and no reinforcement was given. The limited hold and time-out duration systematically varied across stages of training and testing (see below). The sample ball was cleaned and returned as described above within a ~8 s intertrial interval.

#### Sucrose Detection

To begin lick training, the rat had access to one ball per day while the other two were made inaccessible with shutters. This was done to familiarize the animals with the ability to obtain fluid from the sample and each of the two response balls. During these three 30-min sessions the rat had unlimited access to DW by licking on either the center (sample), left (response), or right (response) balls, whichever was available.

Next, the rat was given side training to have it associate one response ball with no tastant (DW) and the other response ball with a tastant (0.6 M sucrose). Across six 30-min sessions, either DW (three sessions) or sucrose (three sessions) was obtained from the center sample ball, and the response was reinforced with DW from the assigned response ball (left or right); the other response ball was blocked with the shutter. Response ball assignments were counterbalanced across groups and an equal number of the rats had the left or right response ball paired with sucrose. The rat was allowed 180 s to respond (i.e. limited hold) and, if there was a correct response, the rat received 20 licks (or 5-s access, whichever came first) of DW from the response ball. During this phase of training, if the rat did not lick the response ball, it would not receive the reinforcer (DW), but there was no time-out.

Then, during the alternation phase of training, in a single session, the rat was presented with DW or 0.6 M sucrose from the sample ball and then chose a response ball. A single stimulus (either DW or 0.6 M sucrose) was repeatedly presented until the rat performed a set number of correct responses (nonconsecutive; criterion decreased over each session from 8, 6, 4, to 2 correct trials) at which point the stimulus was switched. During this phase of training, the limited hold was 15 s and an incorrect or no response was followed by a 20-s time-out.

During Random Training I stimulus deliveries (0.6 M sucrose vs. water) were presented at random and the limited hold was reduced to 10 s. This phase, which allowed for a more gradual change in trial parameters, lasted for two sessions. During Random Training II trials continued to be presented at random and the limited hold was further reduced to 5 s. All of the rats were run in this training phase until they reached a criterion performance of ≥80% correct overall on trials with a response, which occurred after 4 sessions.

During the testing phase rats received successively lower concentrations of sucrose on Tuesdays and Thursdays (referred to as “test sessions”). During control sessions (Monday, Wednesday, and Friday), a given rat received the lowest sucrose concentration with which it consistently performed ≥80%. This was done to ensure that stimulus control was maintained throughout testing by taking into account individual sensitivity and performance. Testing began with 0.6 M sucrose and concentrations were subsequently lowered across sessions by approximately 0.18 to 0.30 log_10_ units until all rats reached chance levels (~50%) by the time 0.0005 M sucrose was tested. At the end of the concentration series for a stimulus, if a rat had not performed ≥80% on the control day immediately prior to a test day, then that test concentration was repeated in order to ensure the rat was under stimulus control.

#### KCl Detection

Following the completion of sucrose testing, rats were given an abbreviated training phase for KCl because the rats were already familiar with the task and the apparatus. Training consisted of 3 sessions conducted with the same parameters as Random Training II described above, wherein 0.6 M KCl replaced sucrose as a stimulus, after which all rats were performing ≥80% overall on trials with a response.

The testing with KCl was similar to that described for sucrose. Testing began with 0.6 M KCl and concentrations were subsequently lowered by approximately 0.27 to 0.4 log_10_ units. All of the rats reached chance levels by the time 0.0005 M KCl was tested.

Although all rats reached chance during testing, some rats performed above chance at the lowest KCl concentrations. So to ensure only chemosensory cues were used to perform the task, we subjected all rats to a water control test (Water Control Test I) at the end of KCl testing. All sample tubes were filled with DW and half were arbitrarily assigned as the “tastant” and the other half were assigned as the “non-tastant”. The test was otherwise identical to a stimulus test session.

#### Quinine Detection

Initially the rats were trained with the same parameters as Random Training II as described above using 0.2 mM quinine (Quinine Training I). This concentration was used for six days, and most rats, including SHAM rats, continued to perform at <80% and were not taking all sample licks. Assuming that the decrease in sampling was possibly due to the aversive nature of the stimulus, we lowered the concentration of quinine to 0.1 mM. After five days of training on 0.1 mM quinine, there was still no improvement. Parameters were then modified to increase the likelihood that quinine was not lingering on the tongue between trials. The number of reinforcement licks allowed was increased from 20 to 30. Likewise, time allowed for reinforcement licks was doubled from 5 to 10 s. The intertrial interval was also increased (to ~14 sec). After seven days of training with these new parameters and 0.1 mM quinine, there was still little improvement. We then increased the quinine concentration to 0.4 mM to test whether we simply did not have a sufficiently salient training stimulus. After 20 days of training, all of the rats were performing at ≥80% correct. Before testing began, the original trial structure parameters (Random Training II) were restored.

Quinine detection testing was identical to sucrose and KCl testing and control sessions were the same as described above (Testing I). However, after 0.003 mM was tested, the assessment of psychometric taste sensitivity functions for quinine was halted, due to suspicions that extraneous cues were being used by some animals to help guide performance in the task. Specifically, performance from all rats did not decrease as quinine concentration was lowered, a hallmark of stimulus control in chemosensory psychophysics [46].

One potential cue was the water source. Throughout training and testing, stimulus concentration solutions were made with DW from a specific laboratory faucet, but the DW used as the sample was taken from a Nalgene container kept in the testing room. Thus, it seemed possible that any differences between the tastes of these DWs could have been detected by the rats, especially after months of training and experience. As such, two separate tests were performed. First, a water control test (Water Control Test 2) was conducted as described above after KCl testing, and used only water from the Nalgene source. The second test used both sources of DW (Water Source Test), in which the left response ball was correct for one source and the right for the other source. The results from the Water Source Test confirmed our suspicion that some animals were using the two different water sources as a cue to perform the task (see Stimulus Control, below). It is important to note that because the rats suspected of using external cues clearly reached chance levels of performance during testing with sucrose and KCl, these animals did not appear yet able to use non-tastant cues to guide their performance during those earlier phases of testing.

We subsequently used water from a single source for all test and control sessions. Training was repeated with 0.4 mM quinine (Training II), and the rats performed well, at ≥80% overall on the first day. Testing then began anew (Testing II) as described above.

Testing II of quinine was identical to that used for sucrose and KCl testing except that quinine concentrations were lowered across test sessions by approximately 0.40 to 0.60 log_10_ units until all of the rats performed at chance levels, which occurred by the 0.00006 mM concentration. Control sessions were the same as described above.

After the conclusion of quinine testing, a final water test was conducted (Water Control Test 3), as described above, to once again confirm that the rats had not been using extraneous cues.

### Histology

At the end of the experiment, each rat was anesthetized with an overdose of a euthanasia agent containing sodium pentobarbital (Euthasol, 390 mg/ml pentobarbital sodium, ≥60 mg/kg, i.p.). The rat was transcardially perfused with 0.1 M PBS followed by 10% buffered formalin. Before the brain was removed from the skull, the head was leveled in a stereotaxic frame and a coronal cut was made through the brain just rostral to lambda to ensure that the brain was blocked on an even plane for sectioning. The brain was then removed from the skull and stored in the same fixative for at least 72 h and then was sliced on a vibratome in the coronal plane at 100 μm. The sections were rinsed briefly in DW first and then in a solution of 3% gelatin and 10% ethyl alcohol and then mounted on gelatin-coated slides. The mounted sections were dried overnight, prior to being conventionally dehydrated, Nissl-stained with thionin, and coverslipped.

### Lesion Analysis

On the basis of the location of taste-responsive neurons and thalamic projections [5–7, 9], the GC is conventionally defined as the dysgranular (DI) and agranular (AI) portions of insular cortex that reside dorsal to the rhinal fissure, medial to claustrum, and approximately +0.2 mm to +2.3 mm anterior to bregma along the AP axis. We also defined the center region of GC lying from +0.6 to +1.8 mm AP relative to bregma as the “core”, a region targeted in electrophysiological studies of taste-responsive neurons in awake rats [50–54].

The lesions were analyzed with the aid of a Leica light microscope (model DMRB; McBain Instruments) along with Neurolucida software (MicroBrightField) by one of two observers blinded to the injection group and behavioral outcome of a given rat. A special lesion mapping system was used to quantify the size, location, and bilateral symmetry of the lesions (see [41]). This system allowed us to divide our area of interest (gustatory cortex and surrounding regions) into subdivisions with a two-dimensional (2D) grid and determine the lesion damage for each subregion independently. The grid consisted of rows based on every 100 μm slice (AP) and columns associated with the ML and DV levels of the insular cortex and surrounding areas [55]. In addition to GC (described above), the grid included the following surrounding areas: granular insular cortex (GI), dorsal to GI (D), ventral to rhinal fissure (V), medial to external capsule (M), and claustrum (C). The borders of our area of interest (D, V, and M) had no atlas-defined anatomical landmarks, so we assigned the width of GC for M and its height for D and V to serve as boundaries. To further refine the analysis, GI, DI, AI, D and V included three medial-lateral subdivisions. All of the rows (AP) and columns (ML) combined represented all areas around and within GC on the 2D map.

Each grid cell was given a lesion score based on a ternary scale (1 = complete lesion in the subregion; 0.5 = lesion in half or more of the subregion; 0 = lesion in less than half of the subregion). Each hemisphere was scored separately.

Approximate AP location of each section was based on several anatomical landmarks and that were deemed distinctive to one brain section and that were easily identifiable (Table 2). The AP distance between landmarks (as stated in the atlas [55]) within the brain was divided by the number of slices between those landmarks in each brain. The estimated thickness of each brain section between those two landmarks was adjusted by that factor to determine its AP coordinate. We added additional landmarks to our earlier lesion analysis mapping method [41] to improve the degree of precision in our representation of lesion location across rats.

**Table 2:** Anatomical landmarks based on the rat brain atlas.

Anteroposterior Atlas Coordinate (mm relative to β)	Anatomical Description
4.2	The section right before layer 1a of piriform cortex (Pir1a) separates from the midline
2.76	Striatum (Cpu) becomes visible in the middle of corpus collosum
2.3	Corpus collosum from both hemispheres meet at the midline: genu of the corpus collosum (gcc)
1.56	Indusium griseum (IG) disappears
0.72	Anterior commissure (aca) is no longer lateral to Lateral Ventricle (LV)
0	One section before aca meets at the midline (approximately at bregma)
-0.48	Bed nucleus (BAC) is most prominent and aca is still bridging the two hemispheres
-1.72	CA3 field of the hippocampus becomes visible
-3.36	Ventral portion of LV appears

**Fig 1:**
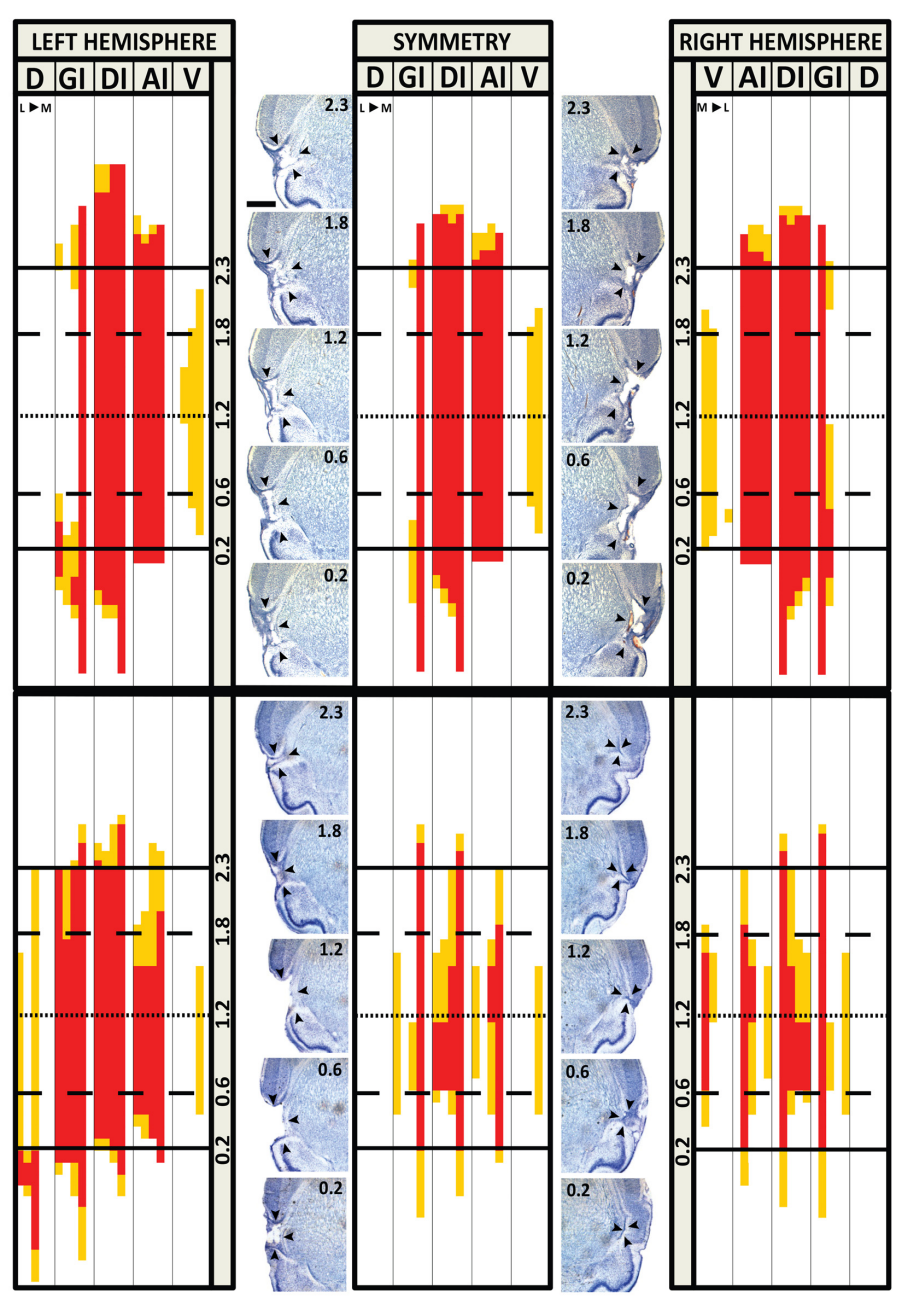
Representative Lesion Symmetry Maps. Top: scoring for a representative large bilateral lesion of a rat whose lesion met the inclusion criteria for behavioral analyses (≥50% of GC, and ≥70% of the GC “core”, containing a lesion). Bottom: scoring for a representative small bilateral lesion in a rat that did not meet the inclusion criteria, and was therefore removed from behavioral analyses. D: Dorsal to granular insular cortex. GI: Granular insular cortex. DI: Dysgranular insular cortex. AI: Agranular insular cortex, dorsal to the rhinal fissure. V: Ventral to the rhinal fissure. L: Lateral. M: Medial. Left and right hemispheres are shown for each representative map along with the resulting Symmetry Map (middle panel). Complete lesion to an area (one grid cell) is indicated in red and received a score of 1.0. Damage less than complete but at least half of the area is shown in orange and was given a score of 0.5. Less than half of the area with destruction, is shown in white and was given a score of 0. The anterior and posterior boundaries of traditionally defined GC are indicated by solid lines at 2.3 and 0.2 mm (AP relative to bregma). Medium-dashed lines show anterior and posterior boundaries of GC “core” at 1.8 and 0.6 mm (AP relative to bregma). The approximate center of GC is indicated with short-dashed lines at 1.2 mm (AP relative to bregma). Photomicrographs of the representative lesion maps are shown with labeled coordinates (in mm, relative to bregma) for each hemisphere. Arrowheads indicate lesion borders. Scale bar = 1 mm.

A symmetry map was created for each rat by comparing each corresponding grid cell of the lesion maps between the left and right hemispheres and conservatively assigning the lesser score to that corresponding grid cell on a separate symmetry grid map (See Fig 1, center panel). If both hemispheres had a score of 1, a score of 1 was assigned; if one hemisphere had a score of 0.5 and the other had a score of 1 or 0.5, a score of 0.5 was assigned. If one hemisphere had a score of 0, no matter if the other hemisphere had a score of 1 or 0.5, a score of 0 was assigned. The sum of the scores in each grid cell in these symmetry maps were then totaled for a designated area (e.g. GC “core”) and used to determine lesion size in GC and outside of GC (regions described above). The proportion of GC with lesion was determined by dividing the total lesion score of GC by the total number of cells within GC (i.e., by the maximum possible score). The proportion of GC “core” with lesion was determined in the same way.

**Fig 2:**
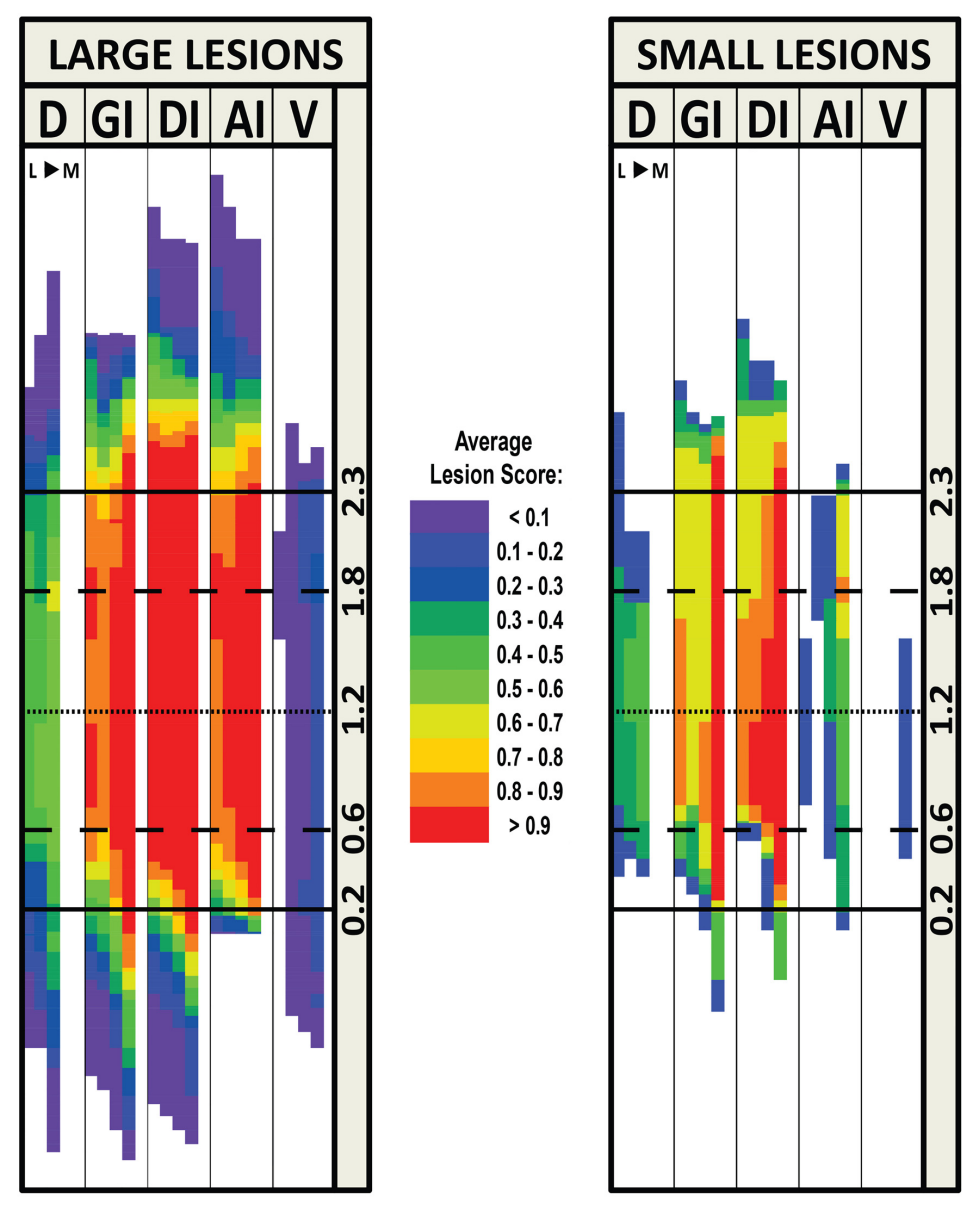
Overlap Map for lesions of rats included in behavioral analyses. Compiled Symmetry Maps of those rats meeting lesion criterion for behavioral analyses (left panel; ≥50% of GC and ≥70% of the GC “core” containing a lesion; n = 26) and lesions too small to be included in behavioral analyses (right panel; n = 3). The lesions for the included rats encompassed 91% of GC and 96% of GC “core” on average. The color key between the maps shows the average lesion score for the group in each cell by color. D: Dorsal to granular insular cortex. GI: Granular insular cortex. DI: Dysgranular insular cortex. AI: Agranular insular cortex, dorsal to the rhinal fissure. V: Ventral to the rhinal fissure. L: Lateral. M: Medial. Solid lines show anterior and posterior boundaries of the traditionally defined GC at 2.3 and 0.2 mm (AP relative to bregma). Medium-dashed lines indicate anterior and posterior boundaries of GC “core” at 1.8 and 0.6 mm (AP relative to bregma). Short-dashed lines indicate the approximate center of GC at 1.2 mm (AP relative to bregma).

The lesion symmetry maps also allowed comparison of average lesion size and location across multiple rats. First, each AP row in the symmetry map for each rat was rounded to the nearest multiple of 10, and then the row was divided into multiple 10 μm rows in order to standardize the AP coordinates across rats. For example, if one row was originally 100 μm of brain thickness, that row would expand into 10 rows of 10 μm. A separate map was then created by averaging each corresponding grid cell of symmetry maps for a defined group of rats (e.g., all rats meeting lesion criteria). The score for each cell is represented in a color scale to depict an overlap map of the lesions (See Fig 2).

### Data Analysis

As in our prior work [40, 42], rats with lesions bilaterally destroying at least 50% of GC and 70% of the “core” were included in the behavioral analysis. As will be shown, most rats had damage that far surpassed these criteria.

The 30-min intake test and the preference scores from the 46-h two-bottle test of the presurgically trained CTA were analyzed with ANOVAs. The intake data from the 46-h two-bottle test was used to calculate CS preference ratios for each rat, in which CS intake was divided by total fluid intake. A ratio close to 1.0 indicates a preference for the CS and a ratio of 0.5 represents equal intake of the CS and DW (i.e., indifference), and a ratio approaching 0 represents CS avoidance.

The brief-access test of the postsurgically trained CTA was analyzed by standardizing the licks to each test stimulus, using a Taste/Water Ratio:

$$Taste/Water\;Ratio=\frac{Mean\;Licks\;to\;Taste\;Stimulus}{Mean\;Licks\;to\;Water}$$

where the mean licks of each rat to a taste stimulus was divided by the mean licks to water in the training session the day before testing. The mean water licks in the test session were unusually low in the LiCl-injected rats, and we suspected that this was possibly due to an aftertaste of the Maltrin, despite the rinse between trials. Thus, the mean licks to water during the prior day’s training session was more representative of each rat’s near-maximal lick rate. All rats took at least 2 trials per concentration of Maltrin. Ratios of 1.0 signify that the licks to the stimulus matched the licks to water, whereas ratios approaching 0 represent avoidance of the taste stimulus. ANOVAs were used to compare Taste/Water ratios across surgical and injection groups. The postsurgical CTA 46-h two-bottle test was analyzed in the same way the presurgical CTA 46-h two-bottle test was, described above.

For sucrose, KCl, and quinine Testing II data, the proportion correct for stimulus and water trials with a response from that session for a single concentration were averaged across control and test sessions for all rats (except those noted below). This was done to adjust for idiosyncratic differences in the number of trials of each stimulus type due to the random schedule of presentation. Data from one control day were not included for rats that were accidentally presented with multiple concentrations of KCl and water instead of just one KCl concentration and water. Only the rats that performed ≥80% on average at the highest test concentration were included in the threshold analysis. Curves were fit to these data using a logistic equation representing performance as a function of concentration:

$$f\left(x\right)=\left[\frac{a-0.5}{\left(1+10^{\left(log_{10}x-c\right)b}\right)}\right]+0.5$$

where x = stimulus concentration, a = asymptotic performance, b = slope, and c = log_10_ stimulus concentration at ½-asymptote (i.e., EC_50_; operationally defined as the detection threshold). These parameters were analyzed using t-tests and average proportion correct was analyzed using two-way ANOVAs. Due to the difficulties in matching concentrations for intensity along the dynamic range for multiple stimuli, we do not directly compare the effects of lesion across the stimuli tested (i.e., using Stimulus as a factor in an ANOVA).

Performance on water control tests was analyzed with one-tailed binomial distribution tests to assure that performance was at chance (i.e., 0.5 correct responses) for each rat. To adjust for those differences in the number of trials of each stimulus type due to the random schedule of presentation, we first calculated an average proportion correct for each stimulus type and then multiplied this average by the total number of trials for use in the binomial distribution test. This helped minimize the influence of response bias on the outcome for the water tests. In all statistical tests, a p ≤ 0.05 was considered significant.

In total, eleven rats died or were removed during the experiment for various reasons including complications from surgery, unexplained weight loss, and abnormal drinking behavior resulting in the reduced sample sizes noted. Data from these rats are included only for those phases that each rat completed. All 44 rat brains were histologically processed and analyzed.

## Results

### Lesion Analysis

Twenty-six GCX rats met our lesion criteria for inclusion in the behavioral analyses. Fig 1 shows a representative map of a bilateral lesion that met the criteria and a bilateral lesion that did not. Among the rats in the GCX group meeting the lesion criteria, on average 91% of the GC and 96% of the GC “core” was destroyed. With such large lesions, damage to areas surrounding GC was unavoidable. Damage was detected in regions medial to GC including claustrum and, in rare cases, areas medial to claustrum, including the most lateral portions of the striatum. In some brains, the lesion extended dorsally and involved the granular insular cortex and somatosensory areas. Occasionally, regions ventral to GC including ventral agranular cortex and the ventral part of claustrum were damaged. Fig 2 presents an overlap map of all 26 lesions that met the criteria. Although there was idiosyncratic damage to brain sites outside of the GC, it can be seen in Fig 2 that in general the lesions were well centered and virtually complete in GC. The 3 rats that were not included in behavioral analyses had on average 39% of the GC and 48% of the core damaged. There was not sufficient uniformity in the size, location, and symmetry of the inadequate lesions to make it useful to compare with animals that had lesions that met the inclusion criteria. An overlap map of these 3 small lesions can also be seen in Fig 2.

### Presurgical and Postsurgical CTA

For the presurgical CTA, as expected, there was no difference in CS intake on the conditioning trial between surgical and injection groups (Tables 3 and 4). The group sizes were: GCX-LiCl, n = 15; GCX-NaCl, n = 14; SHAM-LiCl, n = 7; SHAM-NaCl, n = 7. Although there was a main effect of injection for the 30-min single-bottle intake test, the degree of avoidance in the LiCl-injected rats was low and it was not sustained in the 46-h two-bottle test. While there was no evidence that lesions in the GC compromised CTA expression, as indicated by a lack of a significant surgery x injection interaction on either test (Table 4), the meaning of this is eclipsed by the relative lack of conditioning in the LiCl-injected SHAM control group. The weak aversion was possibly due to the single presurgical conditioning trial that was separated by 105 days from the postsurgical CTA test.

**Table 3:** Mean (SE) for presurgically conditioned taste aversion to NaCl.

	GCX-LiCl	GCX-NaCl	SHAM-LiCl	SHAM-NaCl
Conditioning Trial(intake, mL)	21.07(1.13)	17.58 (0.98)	19.21 (2.36)	18.71 (1.09)
30-min Test(intake, mL)	18.86 (2.16)	24.75 (1.81)	13.21 (3.15)	23.50 (1.10)
46-hr Test(preference)	0.40 (0.05)	0.49 (0.07)	0.40 (0.06)	0.40 (0.08)

**Table 4:** Two-way ANOVAs for presurgically conditioned taste aversion to NaCl.

	Surgery	Injection	Surgery x Injection
Conditioning Trial (intake)	F(1,36) = 0.07, p = 0.80	F(1,36) = 2.03, p = 0.16	F(1,36) = 1.14, p = 0.29
30-min Test (intake)	F(1,36) = 2.22, p = 0.15	**F(1,36) = 12.23, p = 0.001**	F(1,36) = 0.90, p = 0.35
46-hr Test (preference)	F(1,36) = 0.45, p = 0.51	F(1,36) = 0.40, p = 0.53	F(1,36) = 0.56, p = 0.46

Significant effects/interactions indicated in bold.

**Fig 3:**
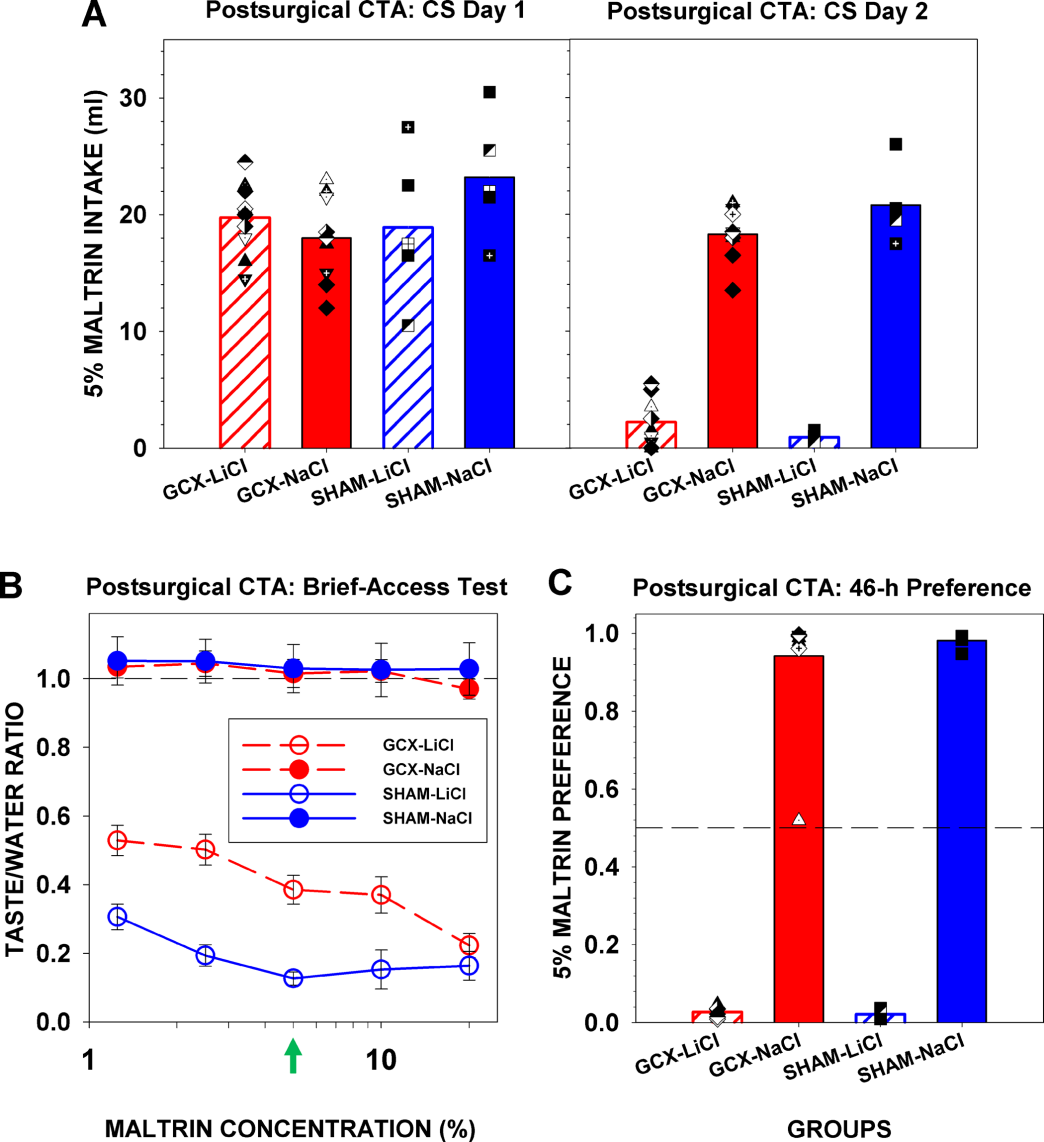
Assessments of a postsurgically conditioned taste aversion to Maltrin. (A) Left panel: Mean (±SE) of CS intake across groups for the first conditioning trial with plotted points indicating individual rats as a function of group. Right panel: Mean (±SE) of CS intake across groups for the second conditioning trial with symbols representing each rat within the group. (B)Taste/Water Ratios for each stimulus by group. A score of 1.0 (dashed line) indicates licking rates comparable to water; a score approaching 0 represents avoidance of the stimulus. The green arrow points to the CS concentration (5% Maltrin). (C) Mean (±SE) group preference scores during the 46-h two-bottle intake test, comparing CS intake to total intake. A score of 1.0 indicates total preference for the CS; a score of 0 indicates total avoidance of the CS. The dashed line at 0.5 represents equal intake of DW and CS. Symbols represent scores for individual animals within the respective groups.

The postsurgical CTA was conducted after taste sensitivity testing. The group sizes were: GCX-LiCl, n = 10; GCX-NaCl, n = 10; SHAM-LiCl, n = 5; SHAM-NaCl, n = 5. Maltrin was used as the CS because it was a novel tastant and, as a polysaccharide, is considered qualitatively discriminable from the stimuli used in the previous tests (i.e. sucrose) [56]. As expected, there was no main effect or interaction involving injection and surgery on CS consumption for the first conditioning trial (Table 5). On the second conditioning trial, intake of all the LiCl-injected animals decreased substantially, indicating a robust aversion, but there was, nonetheless, an injection x surgery interaction likely due in part to the fact that the GCX-LiCl group drank on average twice as much CS compared with the SHAM-LiCl rats (Table 5, Fig 3A).

**Table 5:** Two-way ANOVAs for postsurgically conditioned taste aversion to Maltrin.

	Surgery	Injection	Surgery x Injection
First Conditioning Trial (intake)	F(1,26) = 1.75, p = 0.20	F(1,26) = 0.60, p = 0.45	F(1,26) = 3.38, p = 0.08
Second Conditioning Trial (intake)	F(1,26) = 0.53, p = 0.47	**F(1,26) = 479.18, p<0.001**	**F(1,26) = 5.34, p = 0.03**
46-h Test (preference)	F(1,26) = 0.25, p = 0.62	**F(1,26) = 749.72, p<0.001**	F(1,26) = 0.44, p = 0.51

Significant effects/interactions indicated in bold.

For the brief-access test, a three-way ANOVA (surgery x injection x concentration) of Taste/Water Ratios revealed that the GCX rats showed impaired expression of the CTA as is obvious in Fig 3B and supported by a surgery x injection interaction (Table 6). In addition, a two-way ANOVA of Taste/Water Ratios within the GCX group revealed an interaction between injection and concentration where not all Maltrin concentrations were avoided equally within the GCX-LiCl group likely due to the CS being in the middle of the range of concentrations (Table 7). A two-way ANOVA of Taste/Water Ratios within the injection group showed a significant main effect of surgery for LiCl-injected rats, in support of GC lesion effects on Maltrin licking in the LiCl group (Table 7). All GCX-LiCl rats, except one with a small lesion, had impaired expression of the CTA based on z-scores ≥ 1.96 relative to the mean and standard deviation of the SHAM-LiCl group. Despite showing a clear avoidance on the second conditioning trial, albeit less than the SHAM-LiCl group during a 15-min single-bottle session, when tested across concentrations during the brief-access test, which focuses on the orosensory characteristics of the sampled solution and reduces postingestive feedback and olfactory cues, the GCX-LiCl rats showed a more obvious impairment of CTA expression.

**Table 6:** Three-way ANOVA for a postsurgically conditioned taste aversion to Maltrin in a brief-access test.

	ANOVA
Surgery	**F(1,26) = 6.00, p = 0.02**
Injection	**F(1,26) = 345.99, p<0.001**
Concentration	**F(4,104) = 9.74, p<0.001**
Surgery x Injection	**F(1,26) = 12.85, p = 0.001**
Surgery x Concentration	**F(4,104) = 2.98, p = 0.02**
Injection x Concentration	**F(4,104) = 4.47, p = 0.002**
Surgery x Injection x Concentration	F(4,104) = 1.49, p = 0.21

Significant effects/interactions indicated in bold.

**Table 7:** Two-way ANOVAs for a postsurgically conditioned taste aversion to Maltrin in a brief-access test.

	Concentration	Injection	Concentration x Injection
GCX	**F(4,72) = 12.56, p<0.001**	**F(1,18) = 200.40, p<0.001**	**F(4,72) = 5.56, p = 0.001**
SHAM	**F(4,32) = 3.57, p = 0.02**	**F(1,8) = 136.60, p<0.001**	F(4,32) = 2.00, p = 0.12
	Concentration	Surgery	Concentration x Surgery
LiCl	**F(4,52) = 7.57, p<0.001**	**F(1,13) = 33.83, p<0.001**	F(4,52) = 2.39, p = 0.06
NaCl	**F(4,52) = 2.95, p = 0.03**	F(1,13) = 0.44, p = 0.52	F(4,52) = 0.92, p = 0.46

Significant effects/interactions indicated in bold.

In the two-bottle 46-hr preference test (Fig 3C), there was a main effect of injection, likely due to a strong CTA, resulting from the two conditioning trials administered only 6 days before testing, but no main effect of surgery or interaction between surgery and injection. Thus, in contrast to the brief-access taste test, CTA expression was completely intact in the long-term test.

### Stimulus Control

During the psychophysical testing of taste sensitivity, some issues of stimulus control of behavior were encountered that impacted analyses for those phases and are worth discussing. Although in the past the source and storage of room temperature water has never been a problem, we suspected that water from different containers may have possessed a weak but discriminable cue to some animals during quinine Testing I. This was prompted by a difficulty to find clear-cut decreases in performance as quinine concentration was lowered during the initial testing of this stimulus. Indeed, the results from the Water Source Test using the two water sources suggested that 4 GCX rats, all of which did *not* have monotonically decreasing performance as a function of concentration throughout KCl testing, were using an extraneous cue to perform the task with quinine. Importantly, when Water Control Test 2, using a single water source, was conducted just before the Water Source Test, these rats performed at or near chance levels, implicating the water source rather than a different cue. Although a total of 8 rats performed significantly above chance levels on the Water Source Test, 4 of those rats reached chance levels during testing at the lowest concentrations of KCl and had concentration-dependent responses to quinine during Testing I so that curves were easily fit to the data. However, because it could not be determined exactly when the cue became salient to these animals, data from Testing I for quinine were not included in analyses for any rat.

All but Rat 12 (GCX) performed at chance levels during Water Control Test I, conducted after KCl testing, based on a binomial distribution test, and performance from Rat 12 did not monotonically decrease as a function of concentration for either sucrose or KCl testing. Consequently, the data from Rat 12 were not included in any detection analyses due to the apparent loss of stimulus control throughout testing of taste sensitivity in this animal. Three other rats were not included in the KCl detection analysis because performance was not concentration-dependent during KCl testing despite performing at chance levels during Water Control Test 1.

For Water Control Test 2, although 3 rats performed significantly greater than 50% based on the binomial distribution test, importantly, those rats performed at chance levels at lower concentrations for all of the test stimuli as well as in the preceding and following water control tests (see below) and thus were not suspected of using an external cue to perform the task. Their data were included in analyses.

For the final water control test (Water Control Test 3) after quinine detection Testing II, all rats performed at chance levels, confirming that they needed the chemical stimulus in solution to guide their performance. Unfortunately, 4 rats died after completing quinine detection testing and before the final water control test. However, these 4 rats had monotonic concentration-dependent performance and reached chance levels of responding during testing of the lowest quinine concentrations according to a binomial test and thus were included in the analyses.

### Effects of GC Lesions on Taste Sensitivity

#### Sucrose

**Fig 4:**
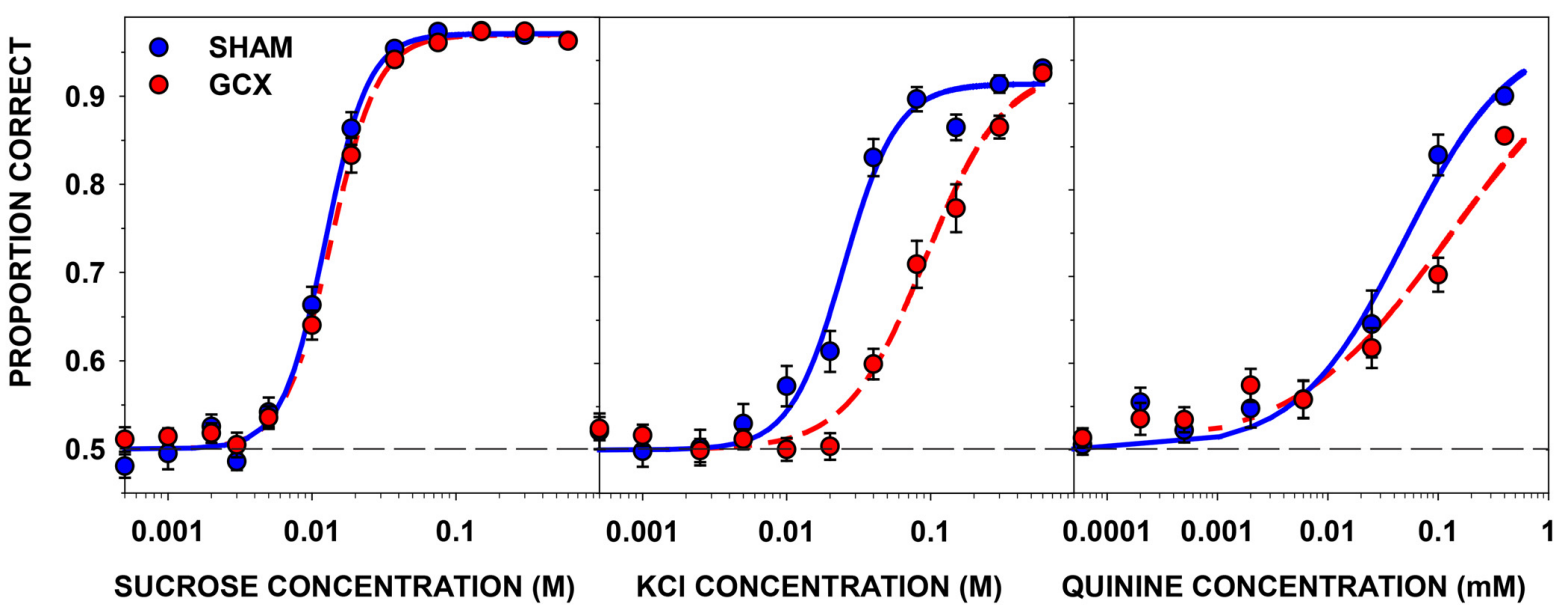
Taste sensitivity testing. Mean (±SE) proportion correct as a function of stimulus concentration for sucrose (left), KCl (center), and quinine (right) sensitivity testing by group. The curves were fit to the data based on a 3-parameter logistic function (see Data Analysis).

**Fig 5:**
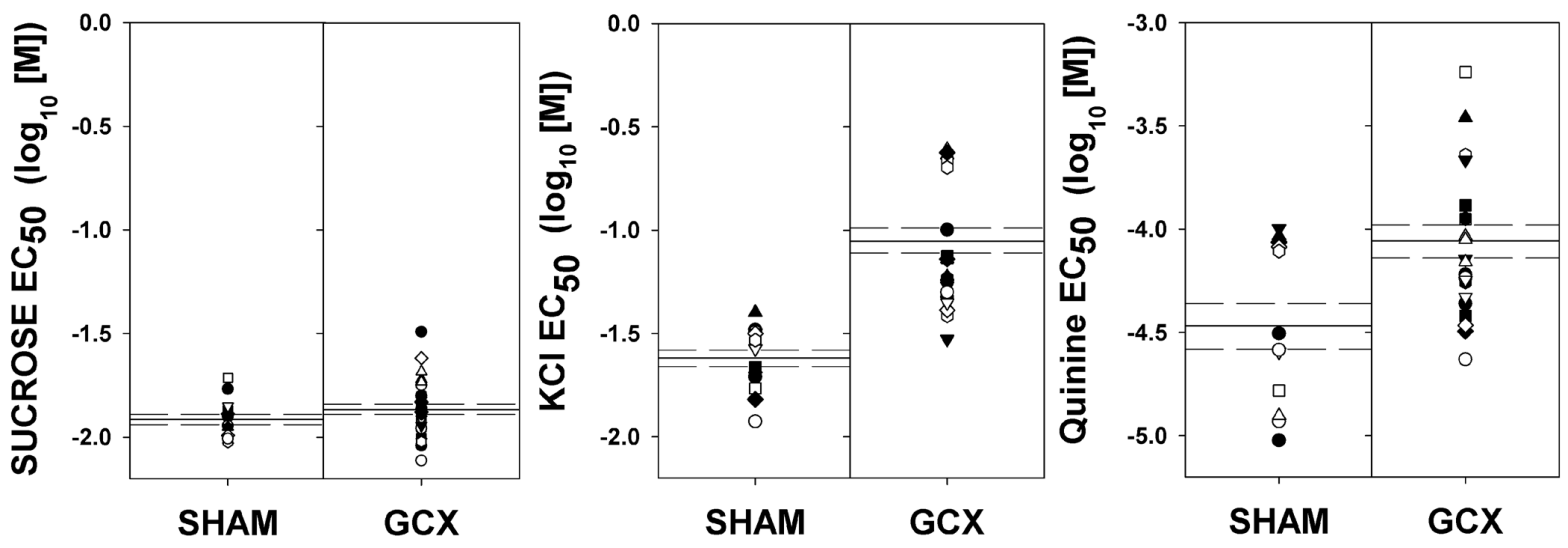
EC_50_ for sucrose, KCl and quinine. Individual EC_50_ values for sucrose (left), KCl (center), and quinine (right). Means are indicated by the solid lines and dashed-lines are the ± SEs. Left panels are SHAM and right panels are GCX rats.

The data for one GCX rat was not included in the analyses due to lack of stimulus control, so the final group sizes for this phase were: GCX, n = 25 and SHAM, n = 13. A two-way ANOVA revealed that there was no main effect of surgery or any surgery x concentration interaction on performance to sucrose. As expected, there was a main effect of concentration (Table 8). The curve fits and the associated EC_50_ values (i.e., c-values) clearly demonstrate the lack of an effect of the GC lesion on sucrose taste sensitivity (Figs 4 and 5). The analyses of the curve parameters between the surgical groups revealed no statistically significant differences for asymptote (a), slope (b), or EC_50_ (c) (Table 9). These results demonstrate that GC is not necessary for normal sucrose detection.

**Table 8:** Two-way ANOVAs for detection testing.

	Surgery	Concentration	Surgery x Concentration
Sucrose	F(1,36) = 0.05, p = 0.83	**F(11,396) = 650.50, p<0.001**	F(11,396) = 1.06, p = 0.40
KCl	**F(1,33) = 36.64, p<0.001**	**F(10,330) = 219.10, p<0.001**	**F(10,330) = 12.63, p<0.001**
Quinine	F(1,33) = 2.7, p = 0.11	**F(7,231) = 122.68, p<0.001**	**F(7,231) = 5.79, p<0.001**

Significant effects/interactions indicated in bold.

**Table 9:** Mean (SE) of curve parameters for detection testing within surgical groups.

	GCX	SHAM		t-test
Sucrose				
a	0.97 (0.003)	0.97 (0.002)		t(36) = 0.94, p = 0.36
b	-3.44 (0.36)	-3.48 (0.31)		t(36) = 0.07, p = 0.95
c^1^	-1.87 (0.03)	-1.91 (0.03)		t(36) = 1.13, p = 0.27
KCl				
a	0.94 (0.01)		0.93 (0.01)	t(33) = 0.59, p = 0.56
b	-3.34 (0.48)		-3.47 (0.94)	t(33) = 0.14, p = 0.89
c^1^	-1.05 (0.06)		-1.62 (0.04)	**t(33) = 6.56, p<0.001**
Quinine				
a	0.96 (0.02)		0.93 (0.01)	t(29) = 1.03, p = 0.31
b	-1.78 (0.45)		-3.51 (1.06)	t(29) = 1.73, p = 0.10
c^1^	-4.06 (0.08)		-4.47 (0.11)	**t(29) = 2.99, p = 0.006**

Significant effects/interactions indicated in bold.

^1^Values in log_10_ (M) concentrations.

#### KCl

The data from 4 GCX rats were not included in the KCl analyses based on water control tests, so the final group sizes for this phase were: GCX, n = 22 and SHAM, n = 13. There was an obvious rightward shift of 0.57 log_10_ units in the KCl psychometric functions representing decreased sensitivity to the salt in the GCX rats compared with the SHAM animals (Figs 4 and 5). A two-way ANOVA revealed both a main effect of surgery and a surgery x concentration interaction on the proportion correct, with performance to lower concentrations obviously more affected by the lesion (Table 8 and Fig 4). The analyses of the curve parameters between the surgical groups revealed statistically significant differences only for the EC_50_ (c) (Table 9 and Fig 4). The lack of difference in the asymptote (a) suggests that both groups were able to perform the task to a similar degree. These data imply that GC is necessary for normal taste sensitivity to KCl.

#### Quinine

Although all of the animals were included in the ANOVA of performance as a function of concentration and surgery, the data for 5 GCX rats were not included in the curve parameter analysis because they did not perform at ≥80% at the highest concentration of quinine (see Methods). The final group sizes for this phase were: GCX, n = 19 and SHAM, n = 12. The GCX rats showed a decrease in sensitivity compared to the SHAM group indicated by a rightward shift of 0.41 log_10_ units (Table 9 and Fig 4). A two-way ANOVA showed a significant main effect of concentration and a significant interaction between surgery and concentration (Table 8). The EC_50_ was significantly higher in the GCX group relative to SHAM rats, but the surgery had no effect of the asymptote or slope of the respective psychometric functions (Table 9 and Fig 5). Overall, these data suggest that GC is necessary for normal quinine detection.

## Discussion

The role of GC in taste-guided behavior is only beginning to be comprehensively understood. Here our experiment revealed GC is necessary to allow rats to normally detect certain taste compounds but not all. In addition to replicating the finding that GC lesions impair KCl sensitivity [42], we found that such lesions also disrupt normal quinine detection in rats. Surprisingly, sucrose sensitivity was not affected by extensive GC damage; apparently central taste processing regions, other than those destroyed here, are sufficient to maintain normal taste detection of this sugar stimulus. Also, the lesions compromised the expression of a CTA to the glucose polymer mixture Maltrin, but only as assessed in specific tests.

### Sensitivity Testing

Although sensitivity to KCl and quinine was impaired by GC damage, the rats were nonetheless able to detect higher concentrations. Clearly, there must be other central gustatory regions besides the GC that contribute to taste detection of the salts KCl and NaCl [42], of the bitter-tasting ligand quinine, and especially of the prototypical sweetener sucrose. The GC has connections with other forebrain areas including the VPMpc, lateral hypothalamus, bed nucleus of the stria terminalis, and the central nucleus of the amygdala [1–15, 17] These forebrain sites receive direct projections from the PBN [16] and thus could potentially maintain some degree of taste detection performance in the absence of the GC. The necessity of various areas of the forebrain gustatory pathways in the maintenance of normal sucrose taste detection could be tested by targeting them alone or in combination, an approach successfully applied, for example, to study the neural substrates of taste aversion learning [57].

Of course, we cannot rule-out the possibility that with even larger lesions, or damage to different regions within insular cortex, sucrose detection would have been impaired. At least one electrophysiological single-unit recording study in rats found that sucrose-best neurons tended to be the most anterior and dorsal of the population of taste-responsive neurons in insular cortex [58]. In an intrinsic imaging study in rats, Accolla et al [10] reported that the center of the area of the neural activation evoked by sucrose was in the insular cortex anterior of the middle cerebral artery. Similarly, using two-photon microscopy of neuronal calcium responses in mouse GC, Chen et al [11] found clusters of neuronal activity evoked by orally administered compounds that were segregated by perceptual taste quality and that the sucrose cluster was most anterior and dorsal. Thus, it may be that the lesions in this study did not adequately damage the area necessary for normal sucrose detection. That said, the successful lesions in this study were large and extended to the anterior border of the GC and beyond, at least as conventionally defined in the rat. Moreover, although we cannot entirely rule out that additional concentrations tested within the dynamic range of the sucrose sensitivity may have revealed a rightward shift in the GCX group, it is clear from the data that the magnitude of such a lesion-induced change would have been necessarily small.

### Presurgical and Postsurgical CTA

It was our intention to determine the effects of the GC lesion on the postsurgical expression of a presurgical CTA to NaCl, but the CTA in the SHAM-LiCl group was weak at best and those rats did not significantly avoid the CS in the 46-h preference test and only weakly did so in the 30-min test. This likely resulted from the administration of only one conditioning trial as well as the extended amount of time between training and testing. We deliberately designed the experiment to include a single conditioning trial so that the CTA would not be robust in hopes that this would increase our ability to detect the effect of the lesion. Unfortunately, the aversion was too weak in the SHAM-LiCl group precluding meaningful assessment of the effect of the GC lesion on this behavior.

The GCX-LiCl rats clearly acquired a postsurgical CTA to Maltrin, though intake was somewhat higher than that of the SHAM-LiCl group on the second conditioning trial in a 15-min single-bottle session. However, when the rats were presented with various concentrations of Maltrin in a brief-access test, noteworthy impairments were evident in the GCX-LiCl group. Yet these same rats, like the SHAM LiCl-injected controls, completely avoided the CS in a subsequent 46-hr two-bottle preference test. The difference in CTA expression between tests is potentially due to the nature of the tasks. There is much more opportunity for postingestive stimulation and olfaction to guide the behavior in the 15-min single-bottle sessions and especially in the 46-hour two-bottle tests as compared with the brief-access test in which small volumes of stimuli are randomly presented and immediate responses are measured [59]. In addition, in the two-bottle test, the animal has water simultaneously available and thus is not forced to consume the CS to achieve hydration.

Previous studies from our lab found significant CTA expression deficits following lesions in and around parts of GC. Schier et al [41, 43] found that it was critical that there be sufficient damage in posterior GC and the overlying visceral cortex in order for impairments in CTA expression as assessed in 15-min single-bottle and 46-hr two-bottle tests to be observed. The lesions in Blonde et al [42] generally included this area and found an effect of the lesion on a presurgical CTA tested postsurgically in a brief-access and preference test. Unlike in the Schier et al studies [41, 43], the lesions in the present study did not lead to impairments in the expression of a postsurgically trained CTA when assessed by a 46-h two-bottle intake test. The lesions in the present study, while including much of the critical region identified by Schier et al [41, 43], were less than complete in their most posterior extents. Alternatively, the basis of the difference might relate to the CS used in the studies. Schier et al [41, 43] used sucrose and NaCl; Blonde et al [42] used sucrose. Whereas here, Maltrin was used. It is possible that the non-gustatory features associated with Maltrin are more salient than sucrose or NaCl in intake tests and that animals were able to take advantage of these cues to guide their conditioned behavior.

There are many factors that could have contributed to the GC lesion-induced deficit in the postsurgically trained CTA expression as assessed by the brief-access test. The lesion could have affected: the saliency of the CS or the US, the memory of the association, the specific expression of the conditioned response, or the establishment of the CS-US association [60]. Our study was not explicitly designed to discriminate among these possibilities. That said, the fact that GCX-LiCl rats showed robust avoidance on the second conditioning trial as well as on the 46-h two-bottle test, suggests that the ability to express, and integrate the CS/US signals did indeed take place, to some degree, and that the stimulus was detectable by some means.

### Final Remarks

There is ample evidence that the area of cortex destroyed here by application of the neurotoxin ibotenic acid normally contains neurons that respond to taste stimulation of the oral cavity [8, 51, 58]. A response of a neuron to a taste stimulus, however, does not, in and of itself, reveal to what function that cell is contributing. Overall, this research supports the notion that the GC is involved in basic sensory discrimination. Thresholds were shifted rightward for KCl and quinine. Importantly, the hedonic characteristics of the stimulus were made irrelevant in the taste detection task used here. That is, the animals were trained to use taste as a cue and thus the responses were not driven by the motivational features of the stimulus. Accordingly, the thalamocortical taste pathway in rats does seem to contribute to basic sensory-discriminative function, with at least some tastants. Nevertheless, the animals with histologically confirmed extensive GC lesions could detect higher concentrations of all of the stimuli tested here, and when sucrose was tested, the animals showed no sign of difficulty detecting this “sweet” stimulus. Obviously, the remaining circuits of the gustatory system are capable of maintaining function in these cases. With the consequences of GC lesions starting to be revealed, it appears that some functions remain completely intact such as taste-related unconditioned hedonic responsiveness and motivation [40, 47] as well as psychophysically assessed sensitivity to sucrose. In contrast, other functions are partially disrupted such as KCl and quinine detection and the rate of acquisition of a NaCl vs. KCl discrimination [42]. Yet other functions are more severely impaired such as CTA expression, provided that the damage includes the entire posterior region of the GC and the overlying granular layer of insular cortex (see [41, 43]).

Given this profile of results, it is tempting to speculate that GC is more involved with higher order taste functions. For example, it has been shown that taste-responsive neurons in the GC begin to respond to auditory cues that signal the delivery of a specific tastant after prior pairings of the two stimuli, suggesting that GC may play a role in processing signals that predict the presentation of particular gustatory stimuli [61]. Perhaps tasks that involve the use of such cues would be disrupted by interference with GC processing [62]. The fact that lesions properly placed in GC and including the adjacent overlying visceral cortex disrupt CTA expression suggests that this portion of insular cortex may be involved in functions that depend on taste-visceral integration and modulate ingestive behavior, but as noted above there are a variety of reasons why a given manipulation might disrupt this form of taste learning. Indeed, a variety of more complex taste tasks might be more vulnerable to GC damage such as the detection of a target stimulus in a mixture or the ability to use short-term memory to compare two tastes delivered over a delay (with an intervening rinse). All of these possibilities share the common denominator of complexity and all are behaviorally testable and await further experimental adjudication.
